# Anterior Nasal Septal Angiomyolipoma Mimicking a Dermoid: A Case Report and Updated Review of an Evolving Sinonasal Entity

**DOI:** 10.7759/cureus.119031

**Published:** 2026-11-02

**Authors:** Faisal Abohelaibah, Abdulmalik Ismail, Mohmmed Alkarzae

**Affiliations:** 1 Otolaryngology - Head and Neck Surgery, Security Forces Hospital, Riyadh, SAU

**Keywords:** angioleiomyoma, angiomyolipoma, dermoid, nasal septum, pecoma

## Abstract

Angiomyolipoma (AML) of the nasal cavity is an uncommon mesenchymal lesion that differs from classic renal and hepatic AML in several clinicopathologic features. We report a 51-year-old man with diabetes mellitus, hypertension, and dyslipidemia who presented with intermittent external nasal swelling and concern about nasal contour, without nasal obstruction or chronic inflammatory sinonasal symptoms. Examination showed a soft, compressible, non-tender anterior nasal lesion. Non-contrast axial computed tomography and magnetic resonance imaging demonstrated a well-defined approximately 2.1 × 2.0 cm lesion in the left anterior nasal cavity/nostril, with a high T2 signal, an internal fatty component, and heterogeneous enhancement, causing displacement of the cartilaginous septum. A dermoid was favored radiologically. The patient underwent septoplasty with excision of the mass in March 2023. Histopathology showed AML with mature adipose tissue, thick-walled vessels, and bland smooth-muscle cells; smooth muscle actin was positive and HMB-45 was negative. Recovery was uncomplicated, with no residual or recurrent lesion on available follow-up. This case highlights an unusual anterior septal location and a radiologic mimic. Recent immunophenotypic and molecular data suggest that many sinonasal lesions historically termed AML are better classified as sinonasal angioleiomyoma with adipocytic differentiation.

## Introduction

Angiomyolipoma (AML) is classically composed of variable proportions of blood vessels, smooth muscle, and adipose tissue. In the kidney and liver, AML belongs to the perivascular epithelioid cell tumor (PEComa) family and commonly shows a myomelanocytic immunophenotype, including smooth-muscle markers together with melanocytic markers such as HMB-45 and Melan-A [1-3]. Nasal AML, first described by Dawlatly et al. in 1988, is much rarer and appears to differ from its renal and hepatic counterpart [1,2]. Most reported sinonasal lesions occur in older adults, show smooth-muscle differentiation, and are not associated with tuberous sclerosis complex (TSC) [2,4,5].

The terminology has become more important with recent pathologic data. In a 2024 multi-institutional study of 15 angiomyolipomatous lesions of the nasal cavity, Jones et al. found no well-developed epithelioid morphology, no melanocytic immunophenotype, and no recurrent pathogenic alterations in TSC1, TSC2, TFE3, or NOTCH2 [5]. The authors proposed the term “sinonasal angioleiomyoma with adipocytic differentiation” for this group of lesions [5]. These related lesions continue to be reported under both AML and angioleiomyoma terminology [6-10].

We present an anterior septal AML that caused intermittent external nasal swelling and septal displacement but was radiologically favored to represent a dermoid. This case is useful because of its unusual anterior location and because it illustrates the evolving classification of sinonasal angiomyolipomatous lesions.

## Case presentation

A 51-year-old man with diabetes mellitus, hypertension, and dyslipidemia was evaluated for intermittent nasal swelling and concern about a change in the external shape of his nose. He denied nasal obstruction and did not report symptoms of allergic rhinitis or chronic rhinosinusitis. Anterior nasal examination showed a soft, compressible, non-tender swelling in the anterior midline/left nasal region, initially thought to be cystic. Septal deviation was also noted. The middle meatus and nasopharynx were clear. There was no documented history of TSC.

On examination, the lesion was located in the left anterior nasal cavity/vestibular region and was attached to the caudal septum. It had a smooth red surface, was soft in consistency, non-tender, and fixed to the caudal septum. It did not extend posteriorly beyond the caudal septal region. A separate measurement on clinical examination was not documented at the initial visit; therefore, we have avoided retrospectively assigning an unverified clinical dimension. The lesion measured approximately 2.1 × 2.0 cm on radiological assessment.

Non-contrast axial computed tomography (Figure 1) and magnetic resonance imaging (Figures 2-4) demonstrated a rounded, well-defined lesion centered within the left nostril/anterior nasal cavity with external protrusion. The lesion measured approximately 2.1 × 2.0 cm, showed high T2 signal intensity, contained an internal fatty component, and demonstrated heterogeneous post-contrast enhancement on MRI. It displaced the cartilaginous septum toward the right, and no other definite facial lesion was identified. A dermoid was favored on the radiology report.

**Figure 1:**
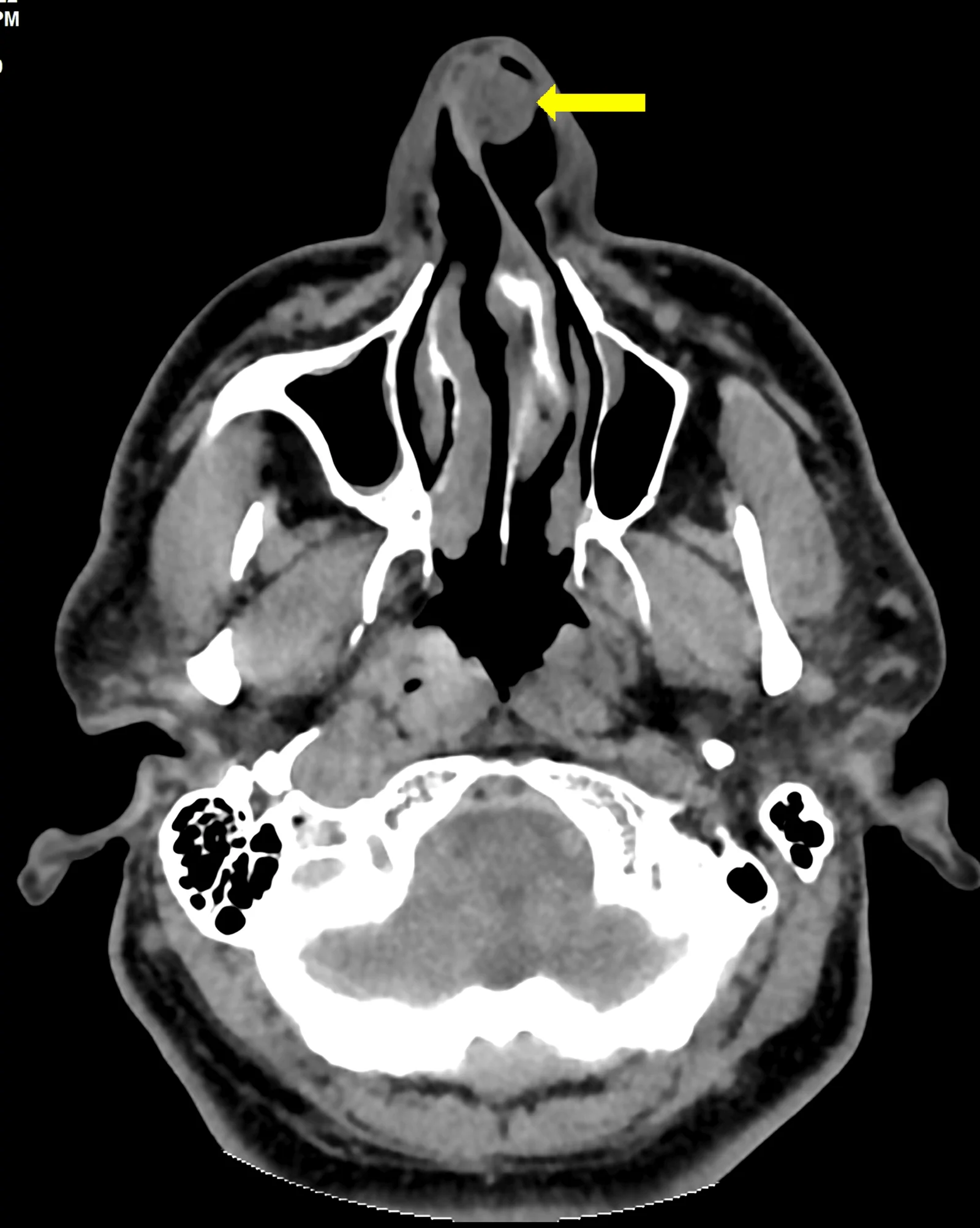
Non-contrast axial computed tomography of the anterior nasal lesion. Non-contrast axial CT demonstrates a well-defined soft-tissue lesion centered in the left anterior nasal cavity/nostril, immediately adjacent to the anterior cartilaginous/caudal septal region, with rightward displacement of the cartilaginous septum. No destructive osseous change is identified.

**Figure 2:**
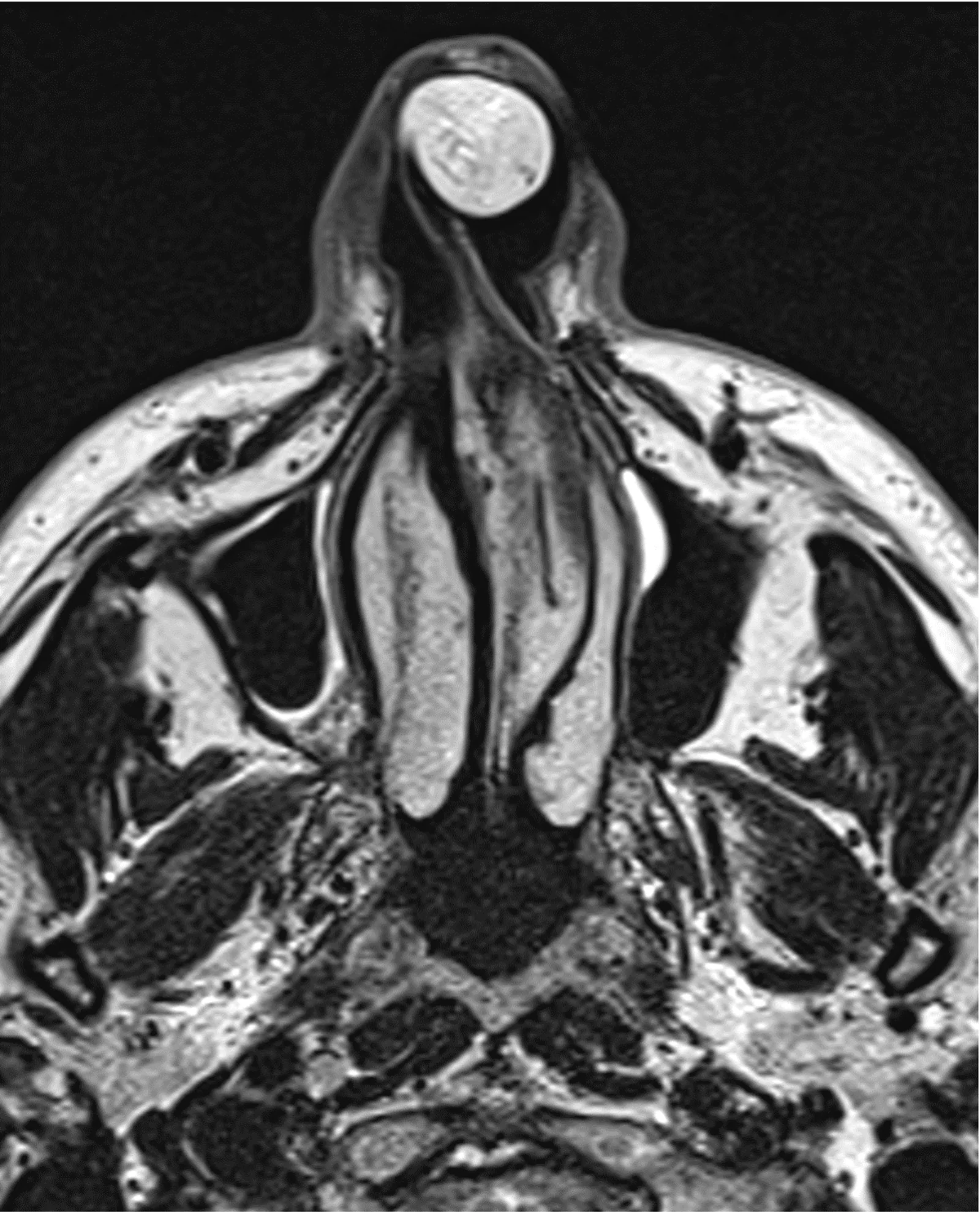
Axial magnetic resonance imaging of the anterior nasal lesion. Axial MRI demonstrates a well-circumscribed lesion in the left anterior nasal cavity adjacent to the anterior/caudal nasal septum. The T2-weighted image demonstrates a high T2 signal with an internal fatty component

**Figure 3:**
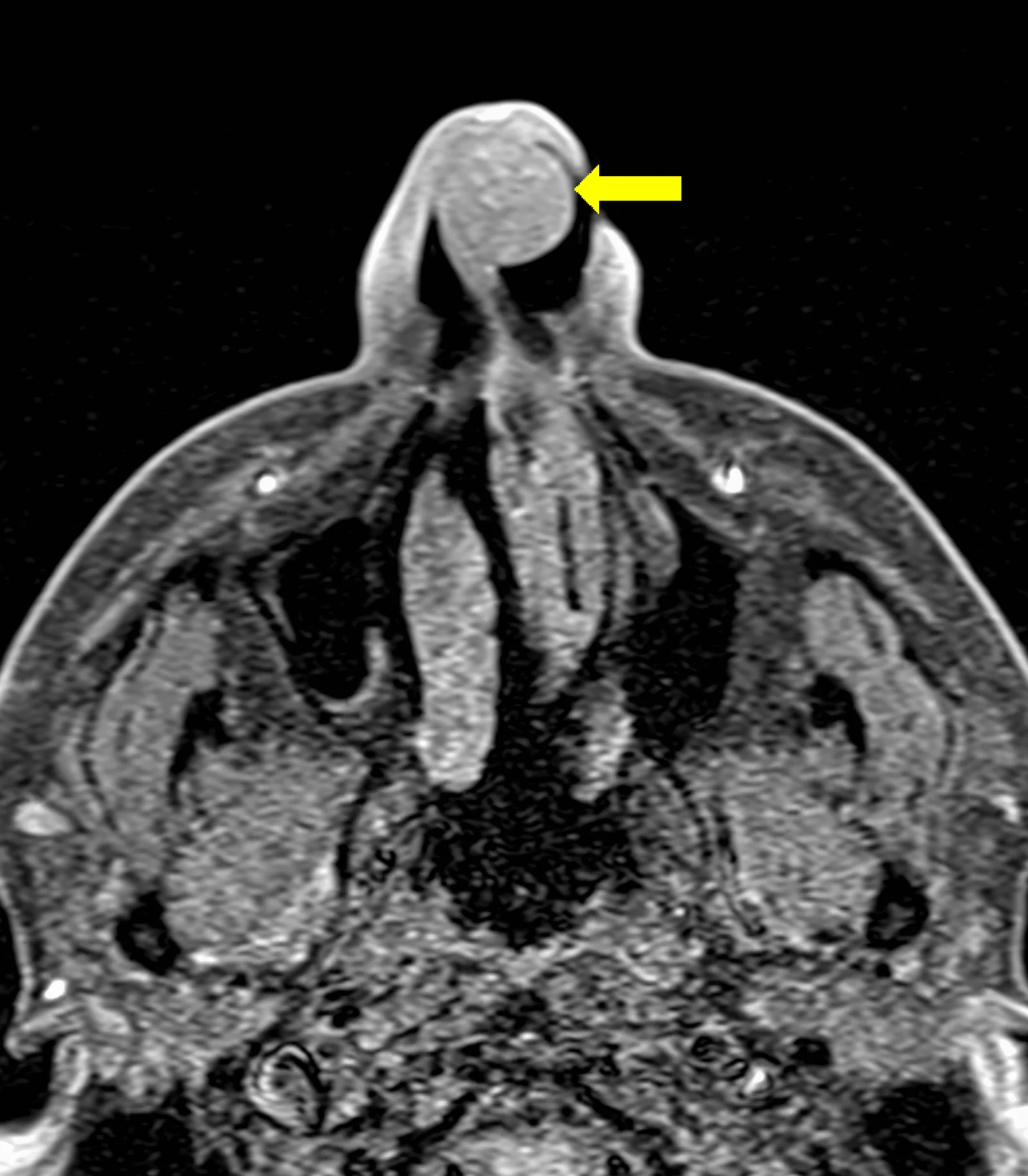
Axial magnetic resonance imaging of the anterior nasal lesion. Axial MRI demonstrates a well-circumscribed lesion in the left anterior nasal cavity adjacent to the anterior/caudal nasal septum. The T1-contrasted image demonstrates heterogeneous enhancement. The lesion produces displacement of the adjacent cartilaginous septum.

**Figure 4:**
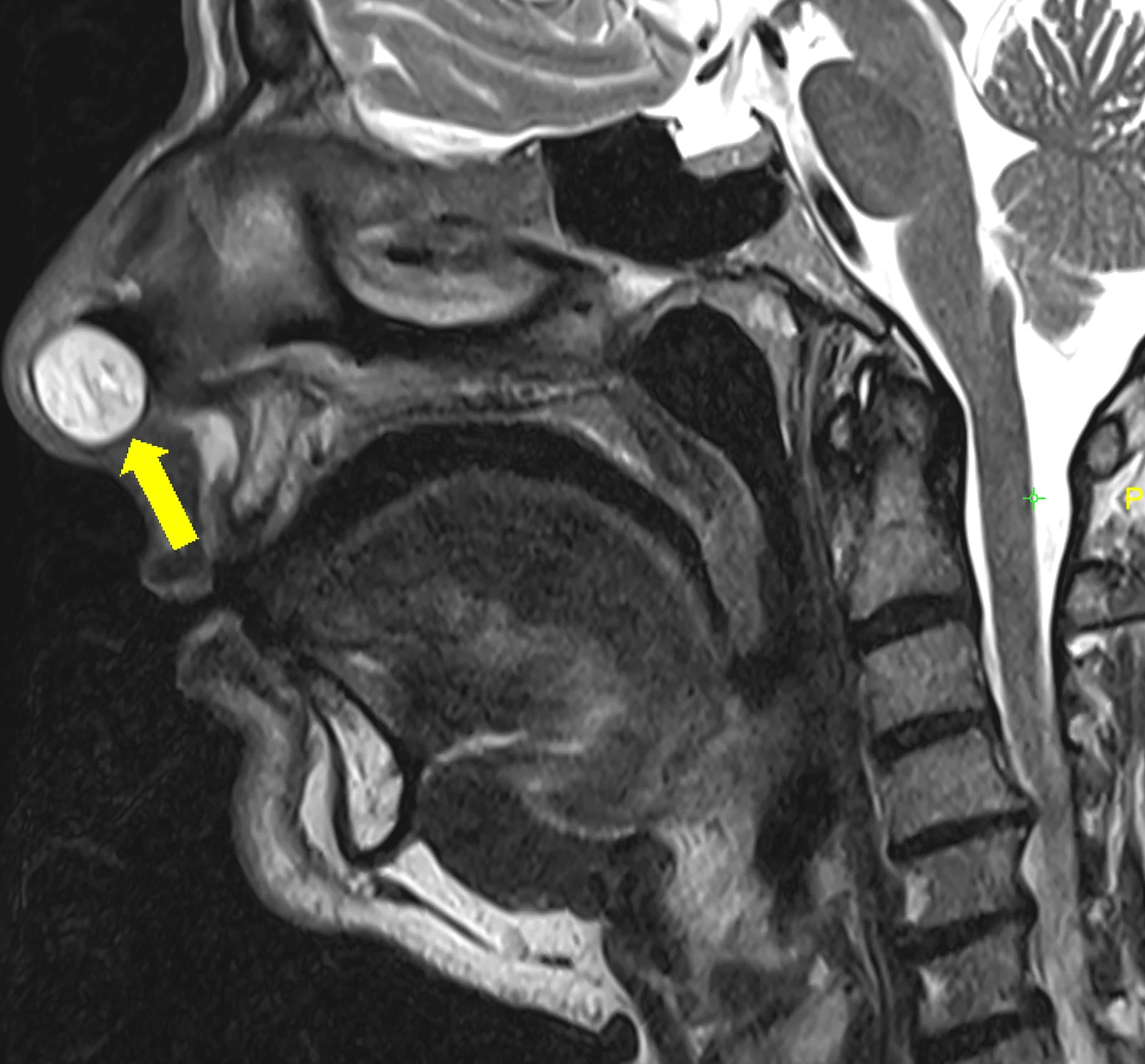
Sagittal magnetic resonance imaging of the anterior nasal lesion. Sagittal MRI demonstrates the markedly anterior position of the lesion at the nasal vestibular/caudal septal region. The T2-weighted image demonstrates its close relationship to the anterior nasal septum and external nasal soft tissues. Intraoperatively, the lesion was confirmed to be attached to the left anterior membranous/caudal septal region.

The patient underwent excision of the intranasal mass with septoplasty. The lesion was described as arising from the left anterior membranous nasal septum. Histopathologic examination established the diagnosis of AML. The lesion was composed of mature adipose tissue, thick-walled vascular channels, and bland spindle smooth-muscle cells. Immunohistochemistry showed smooth muscle actin positivity and negative HMB-45 staining, in keeping with the usual mucocutaneous/sinonasal phenotype rather than classic PEComatous AML.

At the first postoperative visit, the silastic sheet and sutures were removed. Both nasal cavities were patent, the septum was straight, and there was no septal hematoma or perforation. At follow-up, the patient was breathing comfortably and reported no nasal obstruction or epistaxis. Examination showed a clear septum, no residual or recurrent mass, and patent bilateral nasal cavities. He was reassured and discharged.

## Discussion

Sinonasal angiomyolipomatous lesions are rare, and the published evidence consists mainly of case reports and small pathologic series [5,6]. Their clinical pattern differs from that of renal AML. In the 2024 multi-institutional series, the median patient age was 60 years, 87% were male, and none had a history of TSC; obstructive symptoms were the most common presentation [5]. Earlier reviews similarly found that nasal AMLs are usually small, localized lesions without the syndromic association typical of a subset of renal AMLs [4]. Recent reports continue to broaden the clinical spectrum: cases have presented as alar/nasolabial swelling, an inferior-meatal mass with epistaxis, and a vestibular lesion [8-10]. Our patient presented mainly with intermittent external swelling and cosmetic contour change rather than obstruction or bleeding, which is less typical but consistent with the very anterior location of the lesion.

The nasal septum is an unusual site. Liu et al. reported what they described as the first nasal septal AML, arising from the posterior end of the left septum in a 51-year-old woman; the lesion measured 3.5 × 2.7 × 2.9 cm and showed an HMB-45-negative, smooth muscle actin-positive phenotype [7]. In contrast, our lesion was smaller and arose from the anterior membranous septal/vestibular region, where it produced visible external swelling and displacement of the cartilaginous septum. Rather than claiming a definitive “first,” the present case is best regarded as one of the few documented septal angiomyolipomatous lesions and an uncommon example involving the anterior membranous septum. Other unusual sinonasal locations, including the region of the Vidian nerve, have also been reported [11].

Imaging is useful for defining lesion extent and surgical anatomy, but it does not establish the diagnosis. The relative proportions of vascular, smooth-muscle, and adipocytic components vary from case to case and can produce heterogeneous appearances on CT and MRI. Published nasal AMLs have been interpreted preoperatively as hemangioma or juvenile nasopharyngeal angiofibroma [7,12]. In our patient, the anterior location, high T2 signal, internal fat, and heterogeneous enhancement led to a radiologic impression of dermoid. For an anterior nasal mass, the differential diagnosis therefore includes dermoid or epidermoid lesions, lipoma or angiofibrolipoma, lobular capillary hemangioma, angioleiomyoma, other benign vascular tumors, and, when the morphology and immunophenotype support it, PEComa. Imaging should guide assessment of extent and vascularity, while final classification depends on pathology. Additional reports have described lesions presenting with persistent nasal congestion or arising from the nasal vestibule [13,14].

The main pathologic issue is whether lesions historically called nasal AML truly belong to the PEComa family. Watanabe and Suzuki used the term mucocutaneous AML after observing that nasal lesions lacked the epithelioid cells and HMB-45 immunoreactivity typical of PEComatous AML [2]. Wang et al. later argued that nasal AML represents a distinct entity [4]. The 2024 study by Jones et al. provided the strongest evidence to date: all 15 lesions expressed smooth-muscle markers and were negative for melanocytic markers; no well-developed epithelioid morphology was identified; and molecular testing did not show pathogenic alterations in TSC1, TSC2, TFE3, or NOTCH2 [5]. These findings support the proposed designation “sinonasal angioleiomyoma with adipocytic differentiation” and suggest that applying the biologic expectations of renal or hepatic PEComatous AML to most sinonasal lesions may be misleading [5,6].

The classification of the present lesion warrants particular consideration in light of recent pathologic evidence. Although the original diagnosis was AML, based on its triphasic composition of mature adipose tissue, thick-walled vessels, and bland smooth-muscle cells, its SMA-positive and HMB-45-negative immunophenotype differs from that expected in conventional PEComa-family AML. A recent multi-institutional study demonstrated that sinonasal lesions historically classified as AML consistently expressed smooth-muscle markers, lacked melanocytic-marker expression, showed no well-developed epithelioid morphology, and lacked characteristic alterations involving TSC1/TSC2, TFE3, or NOTCH2 [5]. Based on the substantial morphologic overlap with angioleiomyoma and the absence of distinguishing PEComa features, the authors proposed the term “sinonasal angioleiomyoma with adipocytic differentiation” [5]. Our lesion closely fits this reported phenotype, and under current concepts this terminology may therefore be more appropriate than conventional AML. Nevertheless, we have retained “angiomyolipoma” when referring to the original diagnosis because that was the diagnosis rendered at the time of excision. Melan-A was not included in the documented original immunohistochemical panel, and the original histopathology slides were unavailable for contemporary re-review; consequently, definitive retrospective reclassification is limited. Rare monotypic, HMB-45-positive nasal AML has been reported [15], while conventional triphasic nasal AMLs have also been described in earlier literature [16], underscoring the heterogeneity of lesions historically reported under this term.

Complete local excision remains the usual treatment. Endoscopic resection is appropriate for many intranasal lesions, while the approach should be individualized for anterior, septal, or highly vascular tumors [7,11,13,14]. Outcomes are generally favorable. In the multi-institutional series, 11 patients had no recurrence during a median follow-up of 4.7 years, although one angioleiomyoma represented a recurrence six years after a previous procedure [5]. Our patient had an uncomplicated early postoperative course and no recurrence on the available follow-up. The principal limitations of this report are the relatively short documented follow-up and the absence of additional visual pathological and clinical documentation. The original histopathology slides were not available for retrieval, precluding inclusion of H&E and immunohistochemical photomicrographs and contemporary pathological re-review. In addition, preoperative and postoperative clinical photographs were unavailable, limiting objective documentation of the external nasal contour abnormality and postoperative cosmetic outcome.

## Conclusions

This case represents an uncommon anterior septal angiomyolipomatous lesion that presented primarily with external nasal swelling and alteration of the nasal contour rather than nasal obstruction or epistaxis. Imaging demonstrated a well-defined anterior nasal lesion containing a fatty component and showing heterogeneous enhancement, initially favoring a dermoid, highlighting the limitations of radiologic assessment alone in establishing the diagnosis. Histopathology remains essential for definitive characterization.

Although the lesion was originally diagnosed as AML, its SMA-positive and HMB-45-negative immunophenotype, together with the absence of features typical of conventional PEComa-family AML, is more consistent with the recently proposed category of sinonasal angioleiomyoma with adipocytic differentiation. Definitive retrospective reclassification is limited by the unavailability of the original histopathology slides and additional immunohistochemical studies. Complete surgical excision resulted in a satisfactory postoperative outcome with no residual or recurrent lesion during the available follow-up. Further accumulation of well-documented cases with contemporary histopathologic and molecular evaluation will help clarify the most appropriate classification and long-term behavior of these rare sinonasal tumors.
